# The application of existing genotoxicity methodologies for grouping of nanomaterials: towards an integrated approach to testing and assessment

**DOI:** 10.1186/s12989-022-00476-9

**Published:** 2022-05-07

**Authors:** Rachel Verdon, Vicki Stone, Fiona Murphy, Emily Christopher, Helinor Johnston, Shareen Doak, Ulla Vogel, Andrea Haase, Ali Kermanizadeh

**Affiliations:** 1 Nano Safety Research Group, Heriot-Watt University, Edinburgh, UK; 2 Institute of Life Science, Swansea University Medical School, Swansea, UK; 3 National Research Centre for the Working Environment, Copenhagen, Denmark; 4 Department of Chemicals and Product Safety, German Federal Institute for Risk Assessment (BfR), Berlin, Germany; 5 Human Sciences Research Centre, University of Derby, Derby, DE22 1GB UK

**Keywords:** Nanomaterials, Genotoxicity, Grouping, Tiered testing strategy, Alternative physiological multi-cellular models

## Abstract

The incorporation of nanomaterials (NMs) in consumer products has proven to be highly valuable in many sectors. Unfortunately, however, the same nano specific physicochemical properties, which make these material attractive, might also contribute to hazards for people exposed to these materials. The physicochemical properties of NMs will impact their interaction with biological surroundings and influence their fate and their potential adverse effects such as genotoxicity. Due to the large and expanding number of NMs produced, their availability in different nanoforms (NFs) and their utilization in various formats, it is impossible for risk assessment to be conducted on an individual NF basis. Alternative methods, such as grouping are needed for streamlining hazard assessment. The GRACIOUS Framework provides a logical and science evidenced approach to group similar NFs, allowing read-across of hazard information from source NFs (or non-NFs) with adequate hazard data to target NFs that lack such data. Here, we propose a simple three-tiered testing strategy to gather evidence to determine whether different NFs are sufficiently similar with respect to their potential to induce genotoxicity, in order to be grouped. The tiered testing strategy includes simple in vitro models as well as a number of alternative more complex multi-cellular in vitro models to allow for a better understanding of secondary NM-induced DNA damage, something that has been more appropriate in vivo until recently.

**Supplementary Information:**

The online version contains supplementary material available at 10.1186/s12989-022-00476-9.

Over the last two decades, there has been rapid progress in the fields of nanoscience and nanotechnology. The utilisation of nanomaterials (NMs) has offered valuable technological advancements in sectors such as cosmetics, electronics environmental remediation, as well as the development of nanomedicines [1]. Unfortunately, however, the same nano-specific physicochemical properties, which make these materials so unique and attractive, has contributed to concerns about the potential hazards of NMs to human health [2]. These physicochemical properties include but are not limited to small size, high surface area to volume ratio, variation in shape, changes in melting point, solubility or dissolution rate, increased surface reactivity, varying electrical properties and potentially altered crystalline structure of the materials as compared to the bulk form [3]. It is now understood that exposure routes to NMs include ingestion, inhalation, and dermal, with the intravenous route being important for intentional administration of nanomedicines [4]. Recently, a comprehensive battery of *in chemico, *in vitro*, *ex vivo and in vivo studies have demonstrated that NMs can vary in their ability to induce adverse effects including cytotoxicity [5, 6], inflammation [7, 8], autophagy [9, 10], cardiotoxicity [11–15], carcinogenicity [16, 17] and genotoxicity [18–20]. In particular, amongst these associated NM-induced hazards to human health, genotoxicity has attracted much attention due to a causal link to cancer and the potential for inheritable mutations to cause birth defects [21].

The International Agency for Research on Cancer (IARC) categorizes materials and chemicals on their potential carcinogenicity according to the strength of scientific evidence [22]. Carcinogens are natural or synthetic materials that have the potential to cause cancer in living tissues via damaging DNA and chromosomes, inducing aneuploidy, or by disrupting normal cellular metabolic processes. Recently ten key characteristics have been identified with one or more are commonly exhibited for well-established human carcinogens. These characteristics provide the basis for an objective approach to identifying and organizing results from pertinent mechanistic studies. These characteristics are the abilities of a substance to (1) act as an electrophile either directly or after metabolic activation; (2) be genotoxic; (3) alter DNA repair or cause genomic instability; (4) induce epigenetic alterations; (5) induce oxidative stress; (6) induce chronic inflammation; (7) be immunosuppressive; (8) modulate receptor-mediated effects; (9) cause immortalization; and (10) alter cell proliferation, cell death, or nutrient supply [23].

The mechanisms of genotoxicity induced by NMs within a single cell type can be classified into primary (direct and indirect) and secondary damage [24]. During primary genotoxicity, the damage can be elicited by direct interaction of NMs with the genetic content of the cell. This would require the NMs to enter the nucleus and interact directly with DNA. By definition, indirect genotoxicity does not require physical interact of NMs with the DNA, but can be induced by oxidative stress or interactions of mutagens with non-DNA targets leading to damage of proteins involved in DNA replication, cell division, or DNA accurateness. The indirect mechanism of action (MoA) requires either the NMs to deplete antioxidants via promoting ROS production, thus increasing oxidative damage, or to increase oxidative damage via mitochondrial activity. Secondary genotoxicity is classified as ROS damage generated by phagocytes (predominately macrophages and neutrophils) during the NM-induced inflammation causing downstream secondary genotoxicity in other cells [25, 26]. In vivo NM-induced chronic inflammation is associated with a greater risk of secondary genotoxicity of bio-persistent materials and the associated continual generation of ROS and reactive nitrogen species (RNS) causing cell and tissue damage [27].

As touched upon, physicochemical properties of NMs including, shape, size, dissolution, agglomeration state, chemical composition, specific surface area, crystal structure, surface morphology, coating and charge will impact their interaction with biological surroundings, influence their toxicokinetics in the body and their potential adverse effects. As discussed, ROS are key in NM induced primary genotoxicity [28]. Many NMs, and especially carbon-based NMs, are able to generate ROS or RNS in a NM-surface dependent manner [29]. Carbon black-induced genotoxicity to the liver in vivo is likely caused by carbon NM-generated ROS [30]. Additionally, it is now well documented that the small size of such materials and consequent large surface area significantly increases the potential for ROS formation. An enlarged surface area exponentially increases the electroactive sites on the NM, allowing them to be readily exposed. The augmented reaction with molecular oxygen results in the generation of hydrogen peroxide (H_2_O_2_) or superoxide (O_2_^−^) anions [31], which can subsequently oxidize DNA, RNA and other molecules.

Due to the large and expanding number of NMs produced and utilized in various formats and their availability in different nanoforms (NFs) (varying in size, shape, coating etc.), it is recognised that alternative methods are needed to streamline the hazard and risk assessment processes, reducing the need to assess hazard on a case-by-case basis. Such processes will help to make risk assessment and innovation of NFs more financially and ethically viable, as well as more efficient [32]. Alternative approaches such as grouping are suggested by the European Chemicals Agency [33]. Grouping requires a combined demonstration of similarity of physicochemical properties (what they are), toxicokinetics (where they go) and hazards (what they do). The demonstration of similarity by performing grouping allows read across from source substances with available hazard data, to target substances where toxicological data is lacking. The H2020 European project GRACIOUS has developed a Framework to supporting grouping and read across of NFs [34]. The GRACIOUS Framework is underpinned by scientific hypotheses, which identify physicochemical descriptors relevant to grouping of NFs with predicted similar routes of exposure, toxicokinetics and hazard outcomes. Hypotheses for grouping are substantiated by Integrated Approaches to Testing and Assessment (IATA), which encourage analysis of existing information coupled with the generation of new information where needed to support a grouping decision. Each IATA consists of a series of decision nodes that identify the required information, based on the relevant route of exposure, physicochemical descriptors, toxicokinetics and hazards identified in the grouping hypothesis. These decision nodes are structured (e.g. 35), to facilitate efficient decision-making (Fig. 1). If the members of a group are sufficiently similar for the different descriptors identified by the decision nodes, then read-across for regulatory data gap filling can be conducted for a specific hazard endpoint. At least one member from within the group would require sufficient data to meet regulatory requirements for the hazard endpoint of interest (source material), usually in the form of in vivo data. The Framework is designed to be sufficiently sensitive to distinguish between different NFs of the same NM varying in subtle characteristics such as size, crystallinity, functionalisation of the surface of contaminants. At the same time the Framework is sufficiently flexible to allow grouping or comparisons of different NMs of different chemical compositions, which can be useful during the early innovation stages. Case studies to demonstrate these applications have been completed and are being prepared for future publications.

**Fig. 1:**
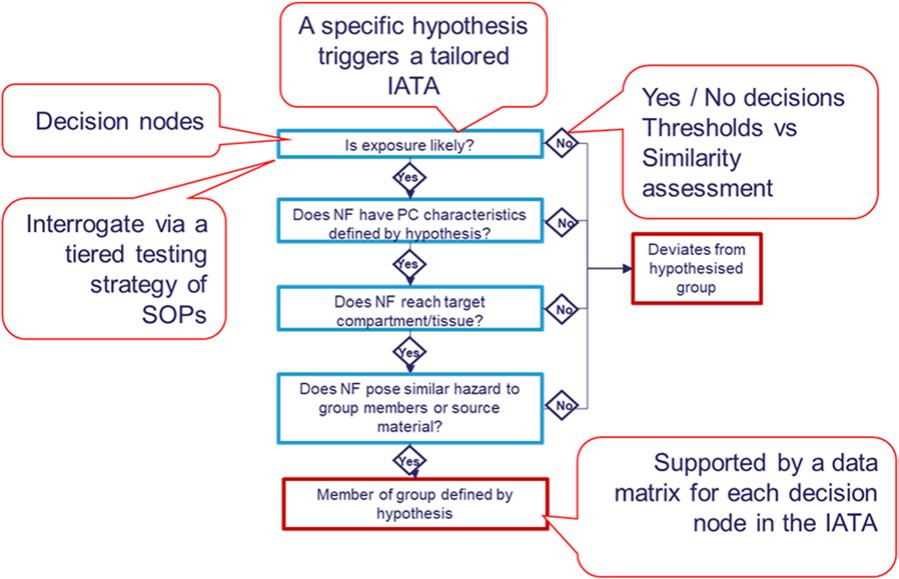
A generic IATA as used in the GRACIOUS Framework to test grouping hypotheses and thereby support grouping of NFs. The blue boxes are the decision nodes which provide the questions to be addressed to streamline the information gathering. Each decision node is supported by a tiered testing strategy consisting of standard operating procedures (SOPs) where possible. Answering all of the decision nodes ‘yes’ results in moving down through the decision tree and acceptance of the hypothesis. If any of the answers is “no” this results in rejection of the grouping hypothesis and exiting the decision tree to the right. The tiered testing strategy described in this short communication would support one decision node in such an IATA

For each decision node, the user is provided with a tiered testing strategy, which identifies the most appropriate methods for gathering the relevant evidence (from existing literature and/or experimentation) required to allow an answer to be generated. For example, for a decision node which addresses the genotoxicity of the NFs within the potential group, the decision node could be worded either ‘Do candidate NFs induce genotoxicity?’ or ‘Are the NFs similar in their ability to induce genotoxicity?’, to which a ‘yes’ or ‘no’ answer could be generated by analysis of the data generated by the tiered testing strategy.

Since the assessment of genotoxicity is relevant for all exposure routes and for many target cell types and therefore for all human health IATAs generated in the GRACIOUS project [35], here, we describe a simple three-tiered testing strategy to assess NF genotoxicity. The tiered testing strategy employs existing genotoxicity testing strategies and methodologies, but places them in a context to support grouping of NFs (Fig. 2). The strategy is based on and builds upon existing guidance for assessment of NF-induced genotoxicity [36–38] and incorporates OECD guidelines when available [39]. In addition and importantly, previous EU projects have also worked and contributed to method adaptation of genotoxicity testing methods required for NFs (e.g. NanoGenoTox, NANoREG) [40]. Figure 2 can be incorporated into any existing human health IATA to allow genotoxicity to be addressed as part of a grouping hypothesis (e.g. respirable, bio-persistent, rigid HARNs—following inhalation exposure and translocation of HARNs to the pleura, mesothelioma development can occur) [35]. In addition, the tiered testing strategy could be used in a new user-defined IATA to address a hypothesis not currently outlined by the GRACIOUS Framework, or it can be used as a stand-alone decision node where the user has a very specific grouping need.

**Fig. 2:**
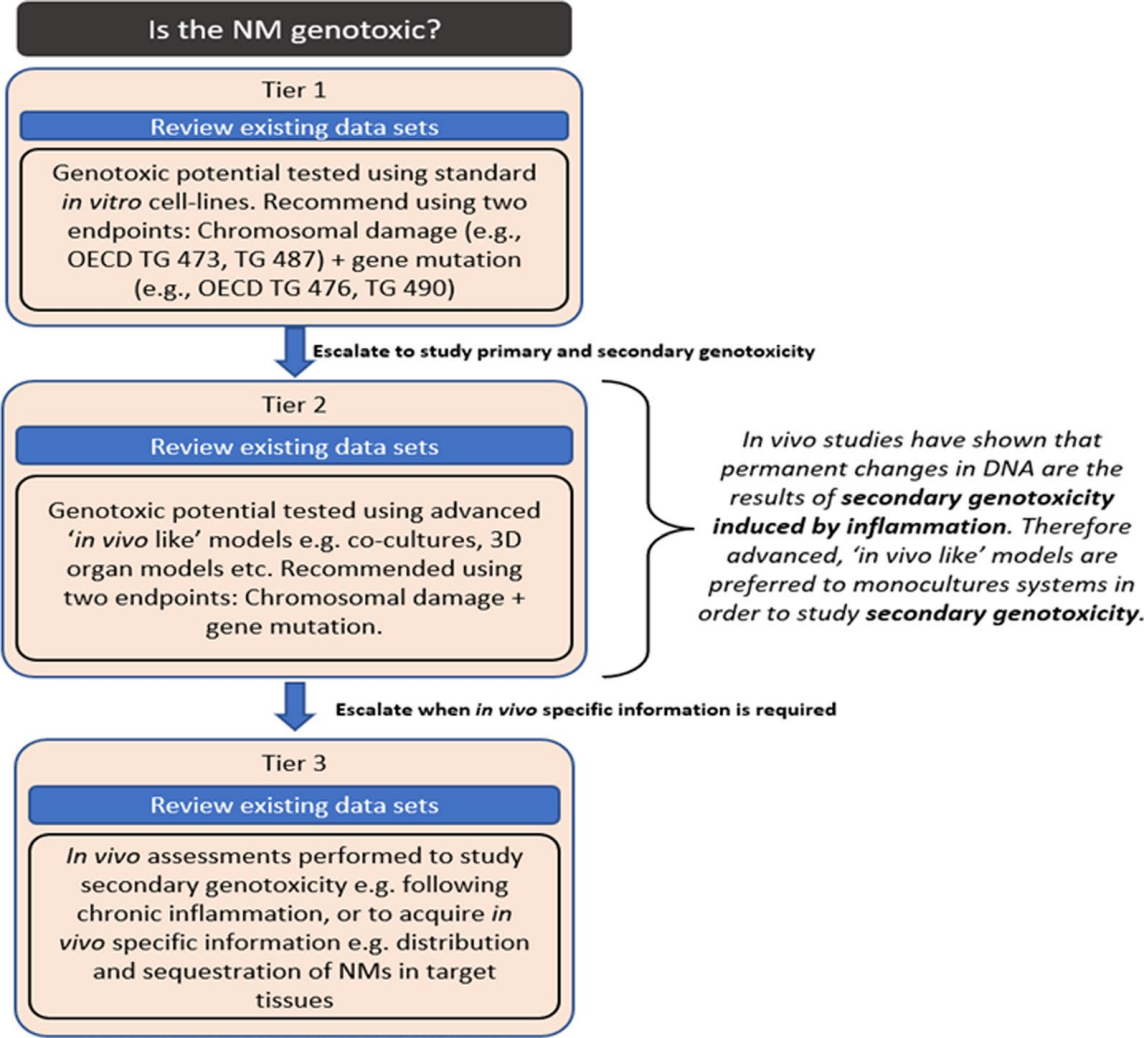
A simple three-tiered strategy to assessing and grouping of NF-induced genotoxicity

For a stand-alone decision node, a simple hypothesis would be required such as ‘NFs with X physicochemical characteristics, following exposure route of Y, would result in genotoxicity in tissues Z’. Acceptance of the hypothesis for each NF under investigation will support the formation of a group. Prior to use of the genotoxicity tiered testing strategy, it is crucial that the NFs are characterized, including a minimum of size distribution, shape, composition (including crystallinity) and surface coating [34]. Further characterization may be required for application of a pre-defined GRACIOUS grouping hypothesis (e.g. dissolution rate in biologically relevant fluids). In addition, information relating to use and exposure scenarios would be needed for NFs to ensure the physicochemical characterization of the NF is relevant to the specific exposure scenario (e.g. in the medium/form of exposure to the body) and resultant target tissues. The characterization methodologies are described elsewhere [34]. Additionally, toxicokinetics information, if available, will also be required to establish which target organs and tissue or cell types are most appropriate for inclusion in the hypothesis. This information may not be required for all candidate NFs in the proposed group, but instead may be read-across from the source NFs/non-NFs to the target NFs where needed. The route of exposure and toxicokinetics information is useful to identify relevant target cell types. Importantly for genotoxicity testing, there is also a requirement for careful consideration for the selection of appropriate cell lines with stable genetic background.

To build a read-across argument all of the target NFs first require either Tier 1 or 2 data to allow an initial assessment of similarity to the source. The same method from the same tier must be used for all NFs for a specific IATA decision node. The similarity assessment may be qualitative (expert judgement) or quantitative [40] and can be used to support read-across to fill the data gaps for the Tier 3 data. If Tier 1 data is utilized, but it is insufficient to support a grouping decision, for example due to variability in data, particle interference with an assay or missing data, then the user may move to Tier 2. The use of Tier 1 data is often sufficient to support decision making relating to potential safety of NFs during the early innovation stages. However, Tier 1 assays will only detect primary genotoxicity potential of a NF. Since most NFs (NFs of very soluble, non-toxic chemicals may be an exception—i.e. nanosized NaCl) have the potential to induce secondary genotoxicity, which requires the presence of immune cells, users may decide to use Tier 2 tests in addition to Tier 1 during innovation.

As stated above, it is important to assess all NFs using the same assay. During the first tier of the proposed testing strategy for the assessment of NF genotoxicity, we recommend the selection of two tests—one for detection of gene mutations (i.e. in vitro mammalian cell gene mutation test or in vitro mammalian mouse lymphoma TK gene mutation assay) and another for chromosomal damage (in vitro micronucleus test, in vitro mammalian chromosomal aberration test) (Fig. 3). Additionally, DNA damage indicator assays such as the comet assay or the Histone H2AX phosphorylation test can be used for detection of NF-induced DNA strand breaks, but these assays are not currently sufficient or accepted for regulatory purposes. Finally, the Ames test is not recommended for NFs due to the fact that bacteria have limited capacity to internalize NFs [37, 38], and because certain NFs have bactericidal properties. It is important that genotoxicity testing is conducted in parallel with cytotoxicity experiments to ensure that an appropriate dose range has been selected as high levels of cell death can confound interpretation of genotoxicity data.

**Fig. 3:**
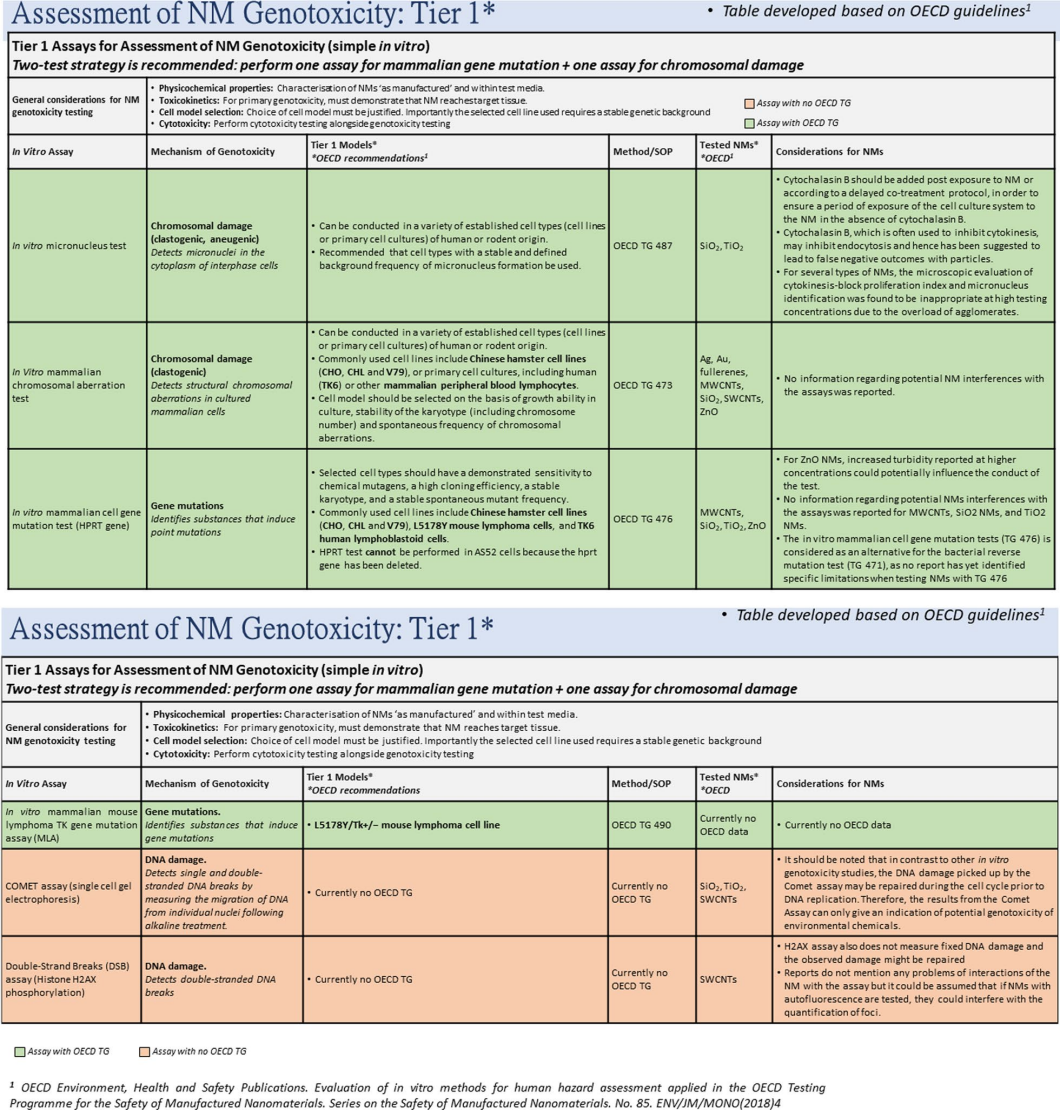
Tier 1 of the strategy for the assessment of NF-induced genotoxicity based on utilisation of simple mono-culture systems and two genotoxicity tests to assess gene mutation and chromosomal damage

Once the tiered testing strategy has been used to assess genotoxicity of the candidate group members, the target and source NFs which produce a positive result in the selected Tier 1 assays (as compared to a well characterised benchmark control for the assay of choice) may be considered qualitatively similar and form a preliminary group. For any NFs which are negative in the Tier 1 assays the grouping hypothesis is rejected and the NF exits the IATA.

Quantitative methods can then be applied to assess similarity in order to identify whether the genotoxicity potential and potency is sufficiently similar to support grouping for regulatory read-across (the MoA will also be important for grouping with respect to genotoxicity). A range of quantitative similarity methods have been described previously [40, 41]. Quantitative methods can either employ pairwise comparisons of NFs in terms of their genotoxicity for each specific assay, or all data for all assays and all NFs can be compared in a multi-comparison analysis using machine learning. The pairwise methods have been shown to be sufficiently robust to use for regulatory grouping and decision making, while the multi-comparison models are less consistent, although they provide potentially useful mechanistic information useful for research purposes. To conduct a similarity assessment, complete high quality data sets [42] are required. A traffic light system to score data completeness and quality has been generated in the GRACIOUS project that can be applied to data uploaded to databases such as eNanoMapper [43]. Furthermore, a review of the existing literature investigating the genotoxicity of nanomaterials (NMs) using in vitro assays recommended in the GRACIOUS Tier 1 and 2 testing strategies has been provided as Additional file 1.

The data from Tier 1 will be very important for allowing a better understanding of the mode of action of genotoxicity and to inform on the selection of the most appropriate Tier 2 assays, if these are required. Crucially, the assays highlighted in Tier 1 do not provide information on secondary NF-mediated genotoxicity, hence the requirement to move to Tier 2 for a better understanding of secondary DNA damage induced by inflammation. Traditional in vitro DNA damage assessment for NFs has heavily relied on single cell mono-cultures. However, in recent years various alternative more complex multi-cellular methods have been developed for the assessment of certain toxicological endpoints that permit or replicate the interaction of different cell types observed in vivo*.* These multi-cellular models can also be utilised for genotoxicity assessment with the aim of allowing better comprehension of secondary DNA damage, something that has only been more feasible in vivo until recently [19, 24, 26]. The development of more complex test systems aim to bridge gaps between in vitro and in vivo NF genotoxicity data.

Once again, testing in Tier 2 is based on the utilisation of two assays, the in vitro micronucleus test, plus one of either the comet assay or the Histone H2AX phosphorylation assay. Significantly, these experiments involve the use of more complex physiologically relevant, multi-cellular test systems (Fig. 4). Where applicable and appropriate, advanced in vitro model protocols are based on SOPs developed in a second European Commission Horizon 2020 funded project, PATROLS (e.g. 44). An additional clear advantage of the use of the more complex Tier 2 models is that they allow for repeated exposure to NFs, which is not always possible with the utilization of the traditional 2D in vitro models. Tier 2 in vitro models, by their design (i.e. 3D spheroids), have longer viability and functionality which can range from days to weeks. Depending on the organ of interest, the route of exposure and material in question this could be highly advantageous. As an example, in the liver, with the exception of nanomedicines, uptake quantities into the body are so low that any potential for “real” NF-induced hazard to man is only likely following long-term repeated exposure. As mentioned above, it is also important to keep in mind that for Tier 2 test models, the selection of appropriate cell types is crucial. As an example, primary hepatocytes do not proliferate in vitro so they would not be suitable for mutagenicity testing; in contrast proliferating cell-lined based hepatocytes can be utilised for this purpose [44]. Finally, it stands to reason that the co-culture models should include immune cells that would be better predictors of NF-induced secondary genotoxicity in vivo.

**Fig. 4:**
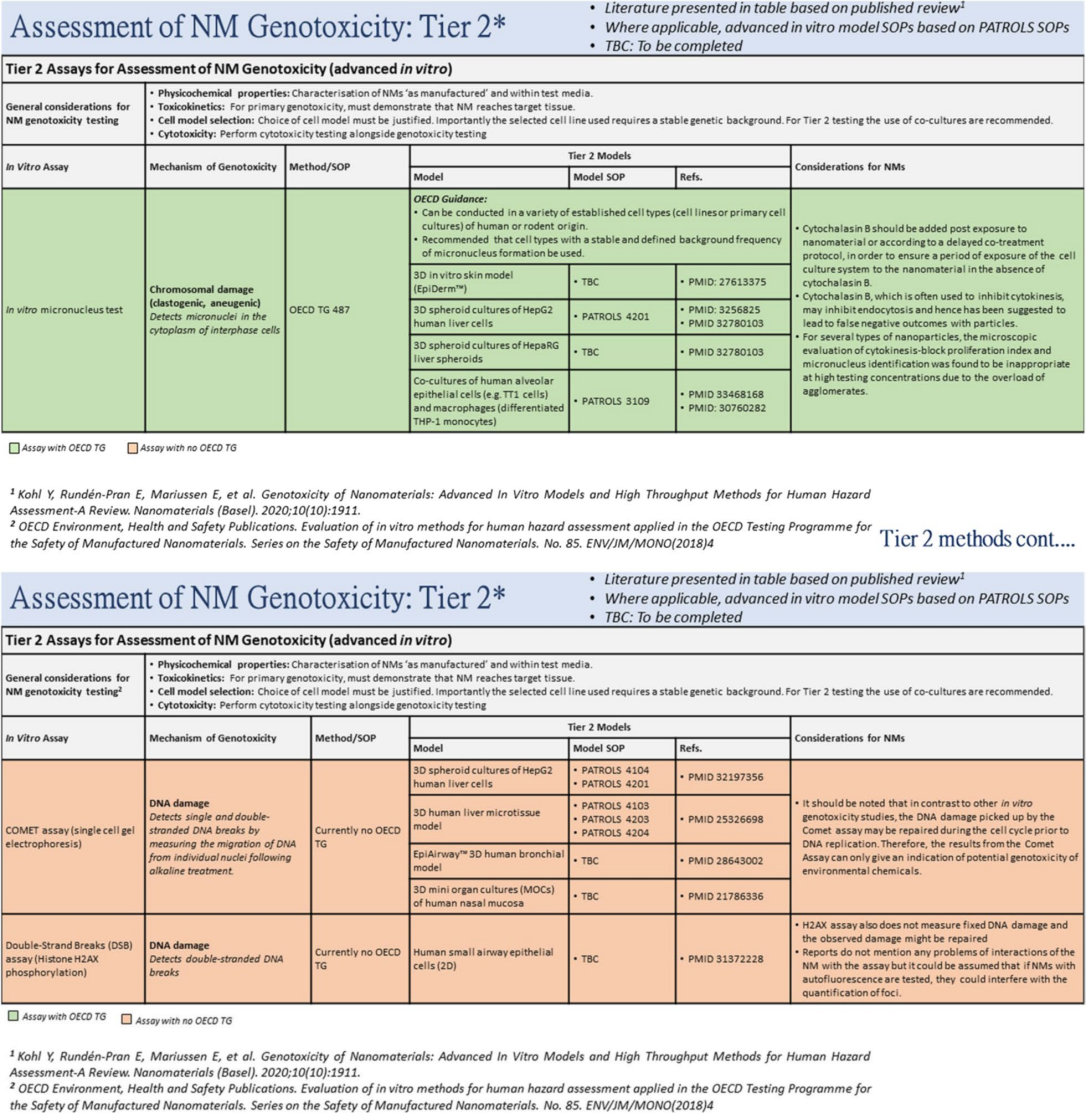
Tier 2 of the strategy for the assessment of NF-induced genotoxicity based on utilisation of advanced co-culture in vitro systems

Progression from Tier 1 to Tier 2 can also be used to strengthen a read-across argument by providing additional mechanistic evidence of similarity in more physiologically-relevant models than the simple in vitro models included in Tier 1. The complex multi-cellular models employed at Tier 2 may be designed based on relevant adverse outcome pathways (AOPs), by, for example, focusing on the activation of specific key events (KEs) or probing key event relationships (KERs, through inclusion of inhibitors etc.) [12, 15]. Demonstration of high levels of similarity in terms of MoA can provide support for the waiving of in vivo toxicity assays, where similarity is based largely on apical toxicity endpoints which may result from divergent MoA. Evidence of in vivo genotoxic consequences to NF exposure may still be required by regulators to validate the predictivity of the in vitro methods employed, as Novel Approach Methods (NAMs) to assess genotoxicity are still under development and validation. However evidence of a high level of similarity between group members could support read-across of existing Tier 3 data from source group members to predict the genotoxic hazard for target group members. If such data is lacking, one member (potentially the worst-case example) can be selected for generation of Tier 3 data.

In summary, in this short communication we propose a simple three-tiered testing approach for grouping of NFs based on their genotoxicity which is in line with the GRACIOUS Framework. The testing strategy can be applied to gather evidence to determine whether different NFs are sufficiently similar with respect to their potential to induce genotoxicity, in order to be grouped. Additionally, in the second Tier of the testing a number of alternative more complex multi-cellular models are suggested as methods to allow for a better understanding of secondary NF-induced DNA damage, something that has only been more feasible in vivo until recently.

## Supplementary Information

**Additional file 1.** A review of the existing literature investigating the genotoxicity of nanomaterials (NMs) using in vitro assays.

## Data Availability

Not applicable.

## References

[1] Gkika DA, Vordos N, Magafas L, Mitropoulos AC, Kyzas GZ (2021). Risk return profile of nanomaterials. J Mol Struct.

[2] Lamon L, Aschberger K, Asturiol D, Richarz A, Worth A (2019). Grouping of nanomaterials to read-across hazard endpoints: a review. Nanotoxicology.

[3] Gupta R, Xie H (2018). Nanoparticles in daily life: applications, toxicity and regulations. J Environ Pathol Toxicol Oncol.

[4] Kermanizadeh A, Powell LG, Stone V (2020). A review of hepatic nanotoxicology—summation of recent findings and considerations for the next generation of study designs. J Toxicol Environ Health Part B.

[5] Belade E, Chrusciel S, Armand L, Simon-Deckers A, Bussy C, Caramelle P, Gagliolo JM, Boyer L, Lanone S, Pairon JC, Kermanizadeh A, Boczkowski J (2015). The role of p53 in lung macrophages following exposure to a panel of manufactured nanomaterials. Arch Toxicol.

[6] Vranic S, Gosens I, Jaconsen NR, Jensen KA, Bokkers B, Kermanizadeh A, Stone V, Baeza-Squiban A, Cassee FR, Tran L, Boland S (2017). Impact of serum as a dispersion agent for *in vitro* and *in vivo* toxicological assessments of TiO_2_ nanoparticles. Arch Toxicol.

[7] Barosova H, Karakocak BB, Septiadi D, Petri-Fink A, Stone V, Rothen-Rutishauser B (2020). An *in vitro* lung system to assess the proinflammatory hazard of carbon nanotube aerosols. Int J Mol Sci.

[8] Kermanizadeh A, Berthing T, Guzniczak E, Wheeldon M, Whyte G, Vogel U, Moritz W, Stone V (2019). Assessment of nanoparticle-induced hepatotoxicity using a 3D human primary multi-cellular microtissue exposed repeatedly over 21 days—suitability of the *in vitro* test system as an *in vivo* surrogate. Part Fibre Toxicol.

[9] Kermanizadeh A, Jantzen K, Ward MB, Durhuus JA, Rasmussen LJ, Loft S, Møller P (2017). Nanomaterial-induced cell death in pulmonary and hepatic cells following exposure to three different metallic materials: the role of autophagy and apoptosis. Nanotoxicology.

[10] Wei M, Le WG (2019). The role of nanomaterials in autophagy. Adv Exp Med Biol.

[11] Hadrup N, Zhernovkov V, Jacobsen NR, Voss C, Strunz M, Ansari M, Schiller HB, Halappanavar S, Poulsen SS, Kholodenko B, Stoeger T, Saber AT, Vogel U (2020). Acute phase responses as a biological mechanism-of-action of (nano)particle-induced cardiovascular disease. Small.

[12] Halappanavar S, van den Brule S, Nymark P, Gaté L, Seidel C, Valentino S, Zhernovkov V, Høgh Danielsen P, De Vizcaya A, Wolff H, Stöger T, Boyadziev A, Poulsen SS, Sørli JB, Vogel U (2020). Adverse outcome pathways as a tool for the design of testing strategies to support the safety assessment of emerging advanced materials at the nanoscale. Part Fibre Toxicol.

[13] Kan H, Pan D, Castranova V (2018). Engineered nanoparticle exposure and cardiovascular effects: the role of a neuronal-regulated pathway. Inhalation Toxicol.

[14] Kuijpers E, Pronk A, Leeman R, Vlaanderen J, Lan Q, Rothman N, Silverman D, Hoet P, Godderis L, Vermeulen R (2018). Cardiovascular effects among workers exposed to multiwalled carbon nanotubes. Occup Environ Med.

[15] Nymark P, Karlsson HL, Halappanavar S, Vogel U (2021). Adverse outcome pathway development for assessment of lung carcinogenicity by nanoparticles. Front Toxicol.

[16] Lison D, van den Brule S, Van Meale FG (2018). Cobalt and its compounds: update on genotoxic and carcinogenic activities. Crit Rev Toxicol.

[17] Sobajima A, Haniu H, Nomura H, Tanaka M, Takizawa T, Kamanaka T, Aoki K, Okamoto M, Yoshida K, Sasaki J, Ajima K, Kuroda C, Ishida H, Okano S, Ueda K, Kato H, Saito N (2019). Organ accumulation and carcinogenicity of highly dispersed multi-walled carbon nanotubes administered intravenously in transgenic ras H2 mice. Int J Nanomed.

[18] Brown DM, Danielsen PH, Derr R, Moelijker N, Fowler P, Stone V, Hendriks G, Møller P, Kermanizadeh A (2019). The mechanism-based toxicity screening of particles with use in the food and nutrition sector via the ToxTracker reporter system. Toxicol In Vitro.

[19] Burgum MJ, Clift MJD, Evans SJ, Hondow N, Taraf A, Jenkins GJ, Doak SH (2021). Few-layer graphene induces both primary and secondary genotoxicity in epithelial barrier models *in vitro*. J Nanobiotechnol.

[20] Llewellyn SV, Niemeijer M, Nymark P, Mone MJ, van de Water B, Conway GE, Jenkins GJS, Doak SH. In vitro three-dimensional liver models for nanomaterial DNA damage assessment. Small. 2021; 17:e2006055 10.1002/smll.202006055 33448117

[21] Ema M, Gamo M, Honda K (2016). Developmental toxicity of engineered nanomaterials in rodents. Toxicol Appl Pharmacol.

[22] Campaign for accuracy in public research. 2021. Accessed 13 May 2021. https://campaignforaccuracyinpublichealthresearch.com/.

[23] Smith MT, Guyton KZ, Gibbons CF, Fritz JM, Portier CJ, Rusyn I, DeMarini DM, Caldwell JC, Kavlock RJ, Lambert PF, Hecht SS, Bucher JR, Stewart BW, Baan RA, Cogliano VJ, Straif K (2016). Key characteristics of carcinogens as a basis for organizing data on mechanisms of carcinogenesis. Environ Health Perspect.

[24] Evans SJ, Clift MJD, Singh N, Wills JW, Hondow N, Wilkinson TS, Burgum MJ, Brown AP, Jenkins GJ, Doak SH (2019). In vitro detection of in vitro secondary mechanisms of genotoxicity induced by engineered nanomaterials. Part Fibre Toxicol.

[25] Akerlund E, Islam MS, McCarrick S, Alfaro-Moreno E, Karlsson HK (2019). Inflammation and (secondary) genotoxicity of Ni and NiO nanoparticles. Nanotoxicology.

[26] Evans SJ, Clift MJD, Singh N, de Oliveira MJ, Burgum M, Wills JW, Wilkinson TS, Jenkins GJS, Doak SH (2017). Critical review of the current and future challenges associated with advanced *in vitro* systems towards the study of nanoparticle (secondary) genotoxicity. Mutagenesis.

[27] Borm PJA, Fowler P, Kirkland D (2018). An updated review of the genotoxicity of respirable crystalline silica. Part Fibre Toxicol.

[28] Paget V, Moche H, Kortulewski T, Grall R, Irbah L, Nesslany F, Chevillard S (2015). Human cell line-dependent WC-Co nanoparticle cytotoxicity and genotoxicity: a key role of ROS production. Toxicol Sci.

[29] Jacobsen NR, Pojana G, White P, Moller P, Cohn CA, Korsholm KS, Vogel U, Marcomini A, Loft S, Wallin H (2008). Genotoxicity, cytotoxicity, and reactive oxygen species induced by single-walled carbon nanotubes and C(60) fullerenes in the FE1-Mutarade mark mouse lung epithelial cells. Environ Mol Mutagen.

[30] Modrzynska J, Berthing T, Ravn-Haren G, Jacobsen NR, Weydahl IK, Loeschner K, Mortensen A, Saber AT, Vogel U (2018). Primary genotoxicity in the liver following pulmonary exposure to carbon black nanoparticles in mice. Part Fibre Toxicol.

[31] Ravanat JL, Cadet J, Douki T (2012). Oxidatively generated DNA lesions as potential biomarkers of *in vivo* oxidative stress. Curr Mol Med.

[32] Prescott MJ, Lidster K (2017). Improving quality of science through better animal welfare: the NC3Rs strategy. Lab Anim.

[33] ECHA recommendations for nanomaterials applicable to the Guidance on QSARs and Grouping of Chemicals. 2016. Accessed 15 April 2021. https://echa.europa.eu/documents/10162/13564/appendix_r6-1_nano_draft_for_committees_en.pdf/cb821783-f534-38cd-0772-87192799b958.

[34] Stone V, Gottardo S, Bleeker EAJ, Braakhuis H, Dekkers S, Fernandes T, Haase A, Hunt N, Hristozov D, Jantunen P, Jeliazkova N, Johnston H, Lamon L, Murphy F, Rasmussen K, Rauscher H, Jimenez AS, Svendsen C, Oomen AG (2020). A framework for grouping and read-across of nanomaterials—supporting innovation and risk assessment. Nano Today.

[35] Murphy F, Dekkers S, Braakhuis H, Ma-Hock L, Johnston H, Janer G, Cristo L, Sabella CS, Jacobsen NR, Oomen AG, Hasse A, Fernandes T, Stone V (2021). An integrated approach to testing and assessment of high aspect ratio nanomaterials and its application for grouping based on a common mesothelioma hazard. Nano Impact..

[36] Braakhuis HM, Murphy F, Ma-Hock L, Dekkers S, Keller J, Oomen AG, Stone V (2021). An integrated approach to testing and assessment to support grouping and read-across of nanomaterials after inhalation exposure. Appl In Vitro Toxicol.

[37] Doak SH, Manshian B, Jenkins GJS, Singh N (2012). In vitro genotoxicity testing strategy for nanomaterials and the adaptation of current OECD guidelines. Mutat Res.

[38] Elespuru R, Pfuhler S, Aardema MJ, Chen T, Doak SH, Farabaugh CS, Kenny J, Manjanatha M, Mahadevan B, Moore MM, Ouedrago G, Stankowski LF, Tanir JY (2018). Genotoxicity assessment of nanomaterials: Recommendations on best practices, assays and methods. Toxicol Sci.

[39] OECD Environment, Health and Safety Publications. Evaluation of *in vitro* methods for human hazard assessment applied in the OECD testing programme for the safety of manufactured nanomaterials. Series on the safety of manufactured nanomaterials, vol. 85. 2018.

[40] Dusinska M, Mariussen E, Rundén-Pran E, Hudecova AM, Elje E, Kazimirova A, El Yamani N, Dommershausen N, Tharmann J, Fieblinger D, Herzberg F, Luch A, Haase A. *In vitro* approaches for assessing the genotoxicity of nanomaterials. In: Nanotoxicity: methods and protocols. 2019. 10.2903/j.efsa.2011.2379. 10.1007/978-1-4939-8916-4_6 30547457

[41] Jeliazkova N, Bleeker E, Cross R, Haase A, Janer G, Peijnenburg W, Pink M, Rauscher H, Svendsen C, Tsiliki G, Zabeo A, Hristozov D, Stone V, Wohlleben W. How can we justify hazard assessment of nanoforms by grouping in order to reduce animal testing? Concepts and usable tools to quantify similarity. 2021; Manuscript in preparation.

[42] Comandella D, Gottardo S, Rio-Echevarria IM, Rauscher H (2020). Quality of physicochemical data on nanomaterials: an assessment of data completeness and variability. Nanoscale.

[43] Basei G, Zabeo A, Rasmussen K, Tsiliki G, Hristozov D (2021). A Weight of evidence approach to classify nanomaterials according to the EU classification, labelling and packaging regulation criteria. NanoImpact..

[44] Llewellyn SV, Conway GE, Shah UK, Evans SJ, Jenkins GJ, Clift MJD, Doak SH (2020). Advanced 3D liver models for genotoxicity testing in vitro following long-term nanomaterial exposure. J Vis Exp.

