# Supplementary material for: The application of existing genotoxicity methodologies for grouping of nanomaterials: towards an integrated approach to testing and assessment
Source: Part Fibre Toxicol. 2022 May 7;19:32. doi: 10.1186/s12989-022-00476-9 (PMC9080165; doi:10.1186/s12989-022-00476-9)
Supplement: Supplementary file 1 — Additional file 1. A review of the existing literature investigating the genotoxicity of nanomaterials (NMs) using in vitro assays. [file 12989_2022_476_MOESM1_ESM.docx]

**Summary of *in vitro* assays recommended in the GRACIOUS tier 1 and tier 2 testing strategy**

A review of the existing literature investigating the genotoxicity of nanomaterials (NMs) using *in vitro* assays recommended in the GRACIOUS Tier 1 and 2 testing strategies was conducted in June 2021 using the search terms and databases outlined in Table 1.

**Table 1** Search dates and keywords investigating genotoxicity of nanomaterials (NMs) using in vitro assays recommended in GRACIOUS Tier 1 and 2 testing strategies

| **No.** | **Date** | **Database** | **Search terms** |
| --- | --- | --- | --- |
| 1 | 10/06/2021 | Pubmed | (nanoparticle* OR nanomaterial* OR nanoform) AND (In vitro micronucle* test OR In vitro micronucle* assay OR OECD TG 487) |
| 2 | 11/06/2021 | Pubmed | (nanoparticle* OR nanomaterial* OR nanoform) AND (In vitro micronucle* test OR In vitro micronucle* assay OR OECD TG 487) |
| 3 | 14/06/2021 | Pubmed | (nanoparticle* OR nanomaterial* OR nanoform) AND (In vitro chromosom* aberration test OR In vitro chromosom* assay OR OECD TG 473) |
| 4 | 14/06/2021 | Pubmed | (in vitro chromosomal aberration test) AND (nanoparticl* OR nanomaterial*) |
| 5 | 14/06/2021 | Pubmed | (in vitro) AND (gamma H2AX) AND (nanoparticl* OR nanomaterial*) |
| 6 | 15/06/2021 | Pubmed | (in vitro) AND (gamma H2AX) AND (nanoparticl* OR nanomaterial*) |
| 7 | 15/06/2021 | Pubmed | (in vitro) AND (hprt) AND (nanoparticl* OR nanomaterial*) |
| 8 | 16/06/2021 | Pubmed | (in vitro) AND (hprt) AND (nanoparticl* OR nanomaterial*) |
| 9 | 16/06/2021 | Pubmed | (in vitro) AND (MLA OR mouse lymphoma assay or mammalian mouse lymphoma TK gene mutation assay OR TG490) AND (nanoparticl* OR nanomaterial*) |
| 10 | 16/06/2021 | WebOfScience | (in vitro) AND (MLA OR mouse lymphoma assay or mammalian mouse lymphoma TK gene mutation assay OR TG490) AND (nanoparticl* OR nanomaterial*) ALL FIELDS |
| 11 | 17/06/2021 | WebOfScience | (in vitro) AND (MLA OR mouse lymphoma assay or mammalian mouse lymphoma TK gene mutation assay OR TG490) AND (nanoparticl* OR nanomaterial*)) ALL FIELDS |
| 12 | 17/06/2021 | WebOfScience | ALL FIELDS: (in vitro) AND (comet assay) AND (nanoparticl* OR nanomaterial*) |
| 13 | 17/06/2021 | Pubmed | (in vitro) AND (comet assay) AND (nanoparticl* OR nanomaterial*) |
| 14 | 18/06/2021 | Pubmed | (in vitro) AND (comet assay) AND (nanoparticl* OR nanomaterial*) |
| 15 | 18/06/2021 | WebOfScience | ALL FIELDS: (in vitro) AND (chromosomal aberration test OR chromosomal assay) AND (nanoparticl* OR nanomaterial*) Refined by: TOPIC: ("chromosomal aberration") |
| 16 | 18/06/2021 | WebOfScience | ALL FIELDS: (in vitro) AND (genotox*) AND (HPRT) AND (nanoparticl* OR nanomaterial*) |
| 17 | 21/06/2021 | WebOfScience | ALL FIELDS: (in vitro) AND (genotox*) AND (HPRT) AND (nanoparticl* OR nanomaterial*) |
| 18 | 21/06/2021 | WebOfScience | ALL FIELDS: (in vitro) AND (genotox*) AND (H2AX or γ-H2AX) AND (nanoparticl* OR nanomaterial*) |
| 19 | 22/06/2021 | WebOfScience | ALL FIELDS: (nanoparticle OR nanomaterial) AND (in vitro micronucleus OR chromosomal aberration OR HPRT OR comet OR H2AX) AND (triple culture OR co-culture) |
| 20 | 22/06/2021 | Pubmed | (nanoparticle OR nanomaterial) AND (in vitro micronucleus OR chromosomal aberration OR HPRT OR comet OR H2AX) AND (triple culture OR co-culture) |
| 21 | 22/06/2021 | Pubmed | (nanoparticle OR nanomaterial) AND (in vitro micronucleus OR chromosomal aberration OR HPRT OR comet OR H2AX) AND (Mn_2_O_3_ OR CuO OR BaSO_4_ OR CeO_2_) |
| 21 | 23/06/2021 | Pubmed | (nanoparticle OR nanomaterial) AND (in vitro micronucleus OR chromosomal aberration OR HPRT OR comet OR H2AX) AND (Mn_2_O_3_ OR CuO OR BaSO_4_ OR CeO_2_) |
| 22 | 24/06/2021 | WebOfScience | ALL FIELDS: (nanoparticle OR nanomaterial) AND (in vitro micronucleus OR chromosomal aberration OR HPRT OR comet OR H2AX OR mouse lymphoma assay) AND (3D) |

The literature search revealed that the most frequently used *in vitro* assays to assess genotoxicity were the *in vitro* micronucleus test (MN) and comet assay (single cell gel electrophoresis). The double stranded breaks assay (also known as Histone H2AX phosphorylation or γ-H2AX assay) has been used often, whereas the mammalian chromosomal aberration (CA) test, mutation test (HPRT gene) and mouse lymphoma TK gene mutation assay (MLA) were less widely used, despite OECD validation. Tests were rarely performed in isolation, commonly run alongside at least one other *in vitro* assay, as well as *in vivo* assays. *In vitro* comet and MN tests were most frequently used together.

The bacterial reverse mutation test (Ames test) was regularly used alongside the recommended mammalian cell assays, despite not being recommended for investigating NMs (OECD, 2020). The literature supports this recommendation, with authors concluding negative results in this assay were due to limited NM uptake into test strains and some bactericidal activity (George et al., 2017, Guo et al., 2016, Li et al., 2012).

The most widely investigated NMs were silver (Ag), followed by titanium dioxide (TiO_2_), and zinc oxide (ZnO). Ag and TiO_2_ have been tested in all the *in vitro* assays recommended in the Tier 1 testing strategy. ZnO and silicon dioxide (SiO_2_), have been tested using multiple assays, whilst carbon nanotubes (CNTs), gold (Au) and copper oxide (CuO) NMs have been investigated to a lesser extent. Iron oxide, cadmium oxide (CdO), Tungsten carbide-cobalt (WC-Co), zirconium dioxide (ZrO_2_), barium sulphate (BaSO_4_) and copper-zinc (Cu-Zn) NMs were investigated less frequently when compared to other NMs. There were discrepancies in the findings between studies with some concluding that specific NMs were genotoxic, whilst other studies did not observe any genotoxicity. It is likely that differences in the findings of different studies are due to variations in the physio chemical properties of NMs (e.g., shape, size, and charge), as well as the experimental design employed e.g., cell type, dose, and exposure time.

**Tiered Testing Strategy**

There was limited evidence assessing the genotoxic potential of NMs performing a chromosomal damage assay and a mutation assay, as recommended in the GRACIOUS tiered testing strategy. Most of the studies used multiple assays to study the effects of NMs on chromosomal damage and assessment of their mutagenic potential has been neglected. Results from studies assessing both endpoints indicate this strategy is beneficial. For example, some studies reported a negative response for chromosomal damage, yet the mutation assay had a positive response (Mei et al., 2012, Mrakovcic et al., 2015) and vice versa (Kim et al., 2010). When Manshian et al. (2013) assessed the genotoxicity of single-walled carbon nanotubes they found that that nanotube length affected genotoxicity, with 400-800 nm nanotubes causing a significant increase in MN formation and 1-3 µm nanotubes causing a significant increase in HPRT mutation.

There is limited research into NM genotoxicity using more complex *in vitro* models, such as 3D models and co-cultures, when compared to monocultures. The available research focuses on the genotoxicity of ZnO, Ag, TiO_2_ and CNTs NMs using more complex *in vitro* models of the skin, lung, liver, and nasal mucosa models. There is an absence of studies which have used gastrointestinal models using the recommended OECD genotoxicity assays. Existing literature commonly used the comet, MN and γ-H2AX assays to assess genotoxicity, however there is a shortage of models that assess mutagenicity. Interestingly, some 3D models indicated that genotoxicity was lower in complex models than in monocultures (Wills et al., 2015).

Existing studies using monocultures have typically used exposure times of 4-24 hours. A benefit of complex models is they allow researchers to measure the effect of longer-term exposures to NMs. For example Kiratipaiboon et al. (2019) assessed the effect CNTs had on human small airway epithelial cells following 6 months of exposure *in vitro*. Other studies have used complex models to examine the effect of repeated NM exposure, allowing recovery periods to assess if NMs cause irreparable damage (Hackenberg et al., 2011b, Kermanizadeh et al., 2014). 3D models often assess fewer exposure times, doses, and endpoints than studies with monocultures of cells.

Co-culture and conditioned media experiments provide evidence of secondary genotoxicity induced by reactive oxygen species (ROS). One study finding that iron oxide NMs were not genotoxic when directly applied to human bronchial epithelial cells (16HBE14o-), however when the epithelial cells were exposed to iron oxide NM treated macrophages (DTHP-1) there was a significant increase in micronucleus formation (Evans et al., 2019). Secondary genotoxicity was also caused by exposure of human bronchial epithelial cells (HBEC) to conditioned medium from macrophages exposed to NiO NMs, with a significant increase of DNA damage in unexposed HBEC cells (Åkerlund et al., 2019).

**Assay Findings**

The **MN assay** was frequently used to assess NM genotoxicity. OECD (2016c) approved cell lines that were commonly used included; human spleen lymphoblast (TK6), human bronchial epithelial cell line (BEAS) and hamster Chinese lung fibroblast (V79). Less frequently used cell lines included OECD approved peripheral blood lymphocytes (PBL), Chinese hamster ovary (CHO), and non-OECD approved murine macrophage (RAW), human glioblastoma (A172) and human neuroblastoma (SHSY5Y). Human liver hepatocellular carcinoma (HepG2), human alveolar basal epithelial adenocarcinoma (A549) and human colorectal adenocarcinoma (Caco2) cell lines were also frequently used however OECD TGs state they are not extensively validated. Exposure times ranged from 3 to 48 hours, few studies assessed more than one exposure time, however several that did report time-dependent responses. Valdiglesias et al. (2013a, 2013b) found no effect of TiO_2_ or ZnO NMs on SHSY5Y cells following a 3-hour treatment, however found a significant dose-dependent increase after 6 hours. In contrast, others observed damage following short term exposure was absent in longer exposures, suggesting DNA repair may have occurred (De Carli, et al., 2018, Llewellyn, et al., 2020). The cytokinesis block proliferation index (CBPI) was frequently used to assess cytotoxicity, with or without additional cytotoxicity tests (Alamar Blue, MTT, XTT, Trypan blue etc.) to ensure MN formation was assessed at sublethal concentrations. Micronucleus formation was assessed using manual counting, cell counting technology and flow cytometry, there was a report that nano-silver may affect the reliability of flow cytometry due to side-scattering and far-red fluorescence (Sahu, et al., 2014). The MN assay has also been used to assess chromosome damage in complex models (Evans et al., 2019, Llewellyn et al., 2020, Wills et al., 2015).

The **comet assay** was also widely used to assess genotoxicity. Studies commonly presented data as ‘percentage of DNA in comet tail’, however some studies presented data on the olive tail moment or tail length. DNA damage was often assessed using a combination of a fluorescent microscope with analysis software (e.g., Comet IV) and has been used to assess genotoxicity in complex models. A study that used the comet test to look at 16 different types of NMs and concluded that as some NMs caused genotoxicity and others did not, the test was competent at assessing genotoxicity (Haase, et al., 2017). The test does not reveal if DNA damage is a result of single or double breaks, some research combines the y-H2AX assay to confirm the presence of damage and identify double stranded breaks. Many studies assessed multiple exposure times which revealed some time-dependent effects. For example, Kazimirova et al. (2019) found an increase of DNA strand breaks in PBLs exposed to a dose of 75 µg/ml TiO_2_ NMs after 4 hours and 15 and 75 µg/ml after 24-hour treatment. Some authors noting reductions in damage during longer exposures may be due to DNA repair mechanisms (Andreoli, et al., 2021, De Carli et al., 2018). Lesion specific enzymes (FPG, Endonuclease III, hOOG1) were used in some studies to assess oxidative DNA damage (Åkerlund et al., 2018, Ávalos et al., 2018, Dorier et al., 2019, Huk et al., 2015, Kazimirova et al., 2019, Mei et al., 2012). There was a noticeable lack of consistency in the cell line used for the comet assay, with few studies using the same type. BEAS and HepG2 were often used, however TK6, CHO, human bronchial epithelial cell (HBEC3), PBL, V79, mouse thymus lymphoblast (L5178Y), RAW, A172, SHSY5Y, A549, Caco2, human Caucasian promyelocytic leukaemia cell (HL60) and adipose-derived mesenchymal stem cells (AMC) were also assessed.

The **γ-H2AX assay** was often used alongside the MN and Comet assays. Cells lines used for this assay also lacked consistency across studies and included HBEC3, PBL, BEAS, A549, Caco2, A172, SHSY5Y, HepG2, renal proximal tubule epithelial cell (HK2), human hepatocyte carcinoma (Hep3B), human renal carcinoma (Caki-1), untransformed human fibroblasts (GM07492), human colon carcinoma (Lovo), and human hepatic adenocarcinoma (Hep1) cell lines. Many studies used software (e.g., CellQuest Pro) to analyse immunofluorescence to quantify double stranded breaks. Others used fluorescence microscopy to image cells and manually assess the presence of γ-H2AX foci formation following fluorescence immunostaining. Many studies use an exposure time of 24 hours; however, times tested range from 1-24 hours. Multiple times assessed often revealed a time-dependent effect for the NM and which also varied with respect to dose. The presence of y-H2AX does not always signify the presence of double stranded breaks (Löbrich et al., 2010), and a second assay is recommended to confirm the presence of DNA damage. Flow cytometry is often used to quantify DNA damage (e.g., precentage of cells staining for γ-H2AX) but researchers have noted than NMs may shield fluorescence (Åkerlund et al., 2018). The γ-H2AX assay has been used to assess DNA damage in complex models (Kiratipaiboon et al., 2019).

The **CA test**, which was used less frequently in published studies, commonly used OECD recommended cell lines; Chinese hamster lung fibroblast (CHL), CHO and PBL. BEAS and AMC were also used. Exposure times ranged from 1 to 72 hours, with some assessing multiple time points, with no significant findings. Metabolic activation was regularly included for CHL cell lines. Assessment of chromosomal aberrations were often subjective, assessed manually through microscopic investigation. A limitation of the test is potential artifact positive results (OECD, 2016a). In the absence of cytotoxicity information chromosomal aberrations are unable to be confidently attributed to the effect of NMs.

The OECD validated **MLA** (OECD, 2016d) was used less frequently to assess NM genotoxicity when compared to other tests. The L5178Y/Tk+/−-3.7.2C cell line was commonly used over TK6 cells. Times tested were often shorter than other assays, around 4 hours, however some studies exposed cells for up to 48 hours. An increase in mutant frequency was a widely used endpoint, not all studies report loss of heterozygosity (LOH) or the increase/decrease of large and small colonies, which can be useful when assessing the type of damage. The OECD guidelines permit the use of agar or microwell versions of the assay, however there was a reported difference in response for Ag NM genotoxicity when using different experimental set ups (Mei et al., 2012)

There were fewer publications covering the ***in vitro* mammalian cell gene mutation test using the HPRT gene**, although it is an OECD validated assay (OECD, 2016b). A commonly used cell line was V79, with some studies using Mus musculus embryonic stem cell (IB10mES), CHO and human lymphoblastoid TK^+/-^ (MCL-5). It has been used alongside comet, MN and y-H2AX assays. Exposure times ranged from 2-48 hours.

***In vivo* Testing**

Some studies included *in vivo* testing, for example *Drosophila melanogaster* (Reis, Rezende et al., 2015), Wistar rats (Kazimirova et al., 2019) and mice models (Wang, al., 2019) alongside *in vitro tests*. There were inconsistencies in the findings, with some supporting the findings from *in vitro* studies (Åkerlund et al., 2018, Ema et al., 2013, Kim et al., 2013, Kwon et al., 2014, Kwon et al., 2014, Landsiedel et al., 2010), and others finding significant genotoxicity *in vitro* studies but not *in vivo* (Ávalos et al., 2018, Chen et al., 2014, Culp et al., 2020, Kazimirova et al., 2019, Wang et al., 2019), and vice versa (Xia et al., 2017).

**Treatments**

Cytotoxicity was regularly assessed alongside genotoxicity to increase confidence, as cytotoxicity can produce artificial positive responses. The OECD TGs recommend testing cells for genotoxicity at sub cytotoxic levels (e.g., less than 55% ± 5% reduction in reduction Relative Population Doubling or Relative Increase in Cell Count). A wide range of methods were used including trypan blue exclusion and alamar blue, LDH, XTT, MTS and MTT assays, and measuring relative population doubling through flow cytometry. However occasionally cytotoxicity levels were not clearly reported, or genotoxicity was assessed at levels of cytotoxicity that exceed OECD TG recommendations.

The selected doses for NM exposure were not always clearly justified and ranged between 0.1 and 2500 µg/ml depending on the study. A few studies noted there were no responses at lower doses and significant responses at higher doses, but the difference between test doses was considerable, creating a gap in the data which could inform if genotoxicity had begun somewhere between the tested values (Reis et al., 2015, Wang et al., 2019). Using a range of doses often revealed dose-dependent responses, however some studies only used one or two doses to assess genotoxicity.

The NM exposure time most frequently assessed was 24 hours. Two exposure times were often compared, and there was limited research with three or more timepoints. Using more than one exposure time often revealed a time-dependent response, with inconsistencies some showing responses in shorter exposures but not in longer exposures (Andreoli et al., 2021, De Carli et al., 2018, Llewellyn et al., 2020) and vice versa (Chen et al., 2014, Kazimirova et al., 2019, Valdiglesias et al., 2013a, Valdiglesias et al., 2013b, Xia et al., 2017). Shorter exposures, around 4 hours were favoured in existing research. Despite OECD recommendations that exposure times should be between 3-6 hours, some research assessed shorter times, e.g., 1- and 2-hour exposures, which often revealed significant genotoxicity (Chen et al., 2014, Hackenberg et al., 2011a, Hackenberg et al., 2011b, Kim et al., 2010, Královec et al., 2019), however comparison with longer exposure times or multiple times could identify potential DNA repair activation and/or time-dependent responses. Multiple time comparisons may also increase confidence that the NM is not genotoxic. There was limited research assessing responses following exposures longer than 48 hours.

Several studies assessed cytotoxicity and genotoxicity in the presence and absence of metabolic activation, which would occasionally impact the results. It was noted that cells tested in serum free conditions may incur increased DNA damage as serum can afford the cells protection (Åkerlund et al., 2018).

**Cells**

For both OECD and non-OECD validated assays there was a wide range of cells employed to assess genotoxicity, and some studies used primary cells. When primary human cells were used donor information was not always published. The OECD recommendation is for human peripheral blood lymphocytes to be obtained from healthy 18-35 years old, who’ve not recently encountered genotoxic agents (OECD 2016c). Donor information was not always provided, or an average age was given, and on occasion ages were outside the OECD recommendations.

Studies that included tests on multiple cell types often noted that some cells were more sensitive to NM exposure than others. Sahu et al. (2014) noted that HepG2 were more susceptible to micronucleus formation than Caco2, with another study finding that Caco2 cells were more susceptible to DNA damage than HepG2 (Abudayyak et al., 2020). Kazimirova et al (2019) suggested that some individuals may be more sensitive than others to TiO_2_ NM exposure when testing peripheral blood lymphocytes. Two studies which examined the genotoxicity of Ag NMs, with similar sizes (42.5±14.5 nm and 46 nm) at a concentration of 10 µg/ml found different results when examining chromosomal aberrations in two cell types. BEAS cells tested for 24 and 48 hours revealed no increase in chromosomal aberrations (Nymark et al., 2013) whereas AMC cells exposed for 1 hour found a significant dose dependent increase (Hackenberg et al., 2011a).

**Reactive Oxygen Species**

ROS production was often analysed alongside the recommended genotoxicity assays. Many of these studies found there to be significant increases in ROS production, with several authors concluding that DNA damage was induced by secondary mechanisms (Åkerlund et al., 2018, Åkerlund et al., 2019, Dorier et al., 2019, Fahmy et al., 2020, Jain et al., 2019, Kumbicak et al., 2014, Kung et al., 2015, Li et al., 2017, Manshian et al., 2013, Paget et al., 2015). Kung *et al.* (2015) noted that poorly differentiated Hep1 cells were more susceptible to NM induced ROS damage, when compared to well differentiated HepG2 cells, due to the lack of a developed antioxidant defence system. Åkerlund et al. (2018) found that ROS generation was significantly increased at higher doses of NiO exposure, but that mutagenicity only increased at the lowest doses. Manshian et al. (2013) found that 89% of oxidative stress response genes were upregulated following exposure to CNTs.

**Conclusions**

Based on the results of the existing literature investigating genotoxicity of nanomaterials, in agreement with the suggested tiered testing strategy, it appears useful to investigate two endpoints (chromosomal damage and mutagenicity). Assessing multiple time points may reveal time dependent genotoxicity, genotoxicity at longer/shorter exposures, DNA repair mechanisms or increase confidence the NM is no genotoxic. Cytotoxicity should be tested in parallel to genotoxicity and not be above OECD TG recommendations, and it is useful to assess a range of doses. The sensitivity of the cell line to NM exposure should be taken into consideration when drawing conclusion regarding NM genotoxicity. Assessing ROS alongside *in vitro* testing using co-culture and conditioned media can provide useful insight into secondary genotoxicity. Complex *in vitro* models have only been tested in a limited number of studies and thus it is recommended that this knowledge gap is addressed in the future.

**References**

Abudayyak M, Guzel E, Özhan G (2020) Cupric Oxide Nanoparticles Induce Cellular Toxicity in Liver and Intestine Cell Lines. *Advanced Pharmaceutical Bulletin* 10: 213-220

Åkerlund E, Cappellini F, Di Bucchianico S, Islam S, Skoglund S, Derr R, Odnevall Wallinder I, Hendriks G, Karlsson HL, Johnson G (2018) Genotoxic and mutagenic properties of Ni and NiO nanoparticles investigated by comet assay, γ‐H2AX staining, Hprt mutation assay and ToxTracker reporter cell lines. *Environmental and molecular mutagenesis* 59: 211-222

Åkerlund E, Islam MS, McCarrick S, Alfaro-Moreno E, Karlsson HL (2019) Inflammation and (secondary) genotoxicity of Ni and NiO nanoparticles. *Nanotoxicology* 13: 1060-1072

Andreoli C, Prota V, De Angelis I, Facchini E, Zijno A, Meccia E, Barletta B, Butteroni C, Corinti S, Chatgilialoglu C, Krokidis MG, Masi A, Condello M, Meschini S, Di Felice G, Barone F (2021) A harmonized and standardized in vitro approach produces reliable results on silver nanoparticles toxicity in different cell lines. *Journal of applied toxicology*

Ávalos A, Haza AI, Mateo D, Morales P (2018) In vitro and in vivo genotoxicity assessment of gold nanoparticles of different sizes by comet and SMART assays. *Food and chemical toxicology* 120: 81-88

Chen Z, Wang Y, Ba T, Li Y, Pu J, Chen T, Song Y, Gu Y, Qian Q, Yang J, Jia G (2014) Genotoxic evaluation of titanium dioxide nanoparticles in vivo and in vitro. *Toxicology letters* 226: 314-319

Culp EJ, Waglechner N, Wang W, Fiebig-Comyn AA, Hsu Y-P, Koteva K, Sychantha D, Coombes BK, Van Nieuwenhze MS, Brun YV, Wright GD (2020) Evolution-guided discovery of antibiotics that inhibit peptidoglycan remodelling. *Nature* 578: 582-587

De Carli RF, Chaves DdS, Cardozo TR, de Souza AP, Seeber A, Flores WH, Honatel KF, Lehmann M, Dihl RR (2018) Evaluation of the genotoxic properties of nickel oxide nanoparticles in vitro and in vivo. *Mutation research Genetic toxicology and environmental mutagenesis* 836: 47-53

Dorier M, Tisseyre C, Dussert F, Béal D, Arnal M-E, Douki T, Valdiglesias V, Laffon B, Fraga S, Brandão F, Herlin-Boime N, Barreau F, Rabilloud T, Carriere M (2019) Toxicological impact of acute exposure to E171 food additive and TiO2 nanoparticles on a co-culture of Caco-2 and HT29-MTX intestinal cells. *Mutation research Genetic toxicology and environmental mutagenesis* 845: 402980-402980

Ema M, Imamura T, Suzuki H, Kobayashi N, Naya M, Nakanishi J (2013) Genotoxicity evaluation for single-walled carbon nanotubes in a battery of in vitro and in vivo assays. *Journal of applied toxicology* 33: 933-939

Evans SJ, Clift MJD, Singh N, Wills JW, Hondow N, Wilkinson TS, Burgum MJ, Brown AP, Jenkins GJ, Doak SH (2019) In vitro detection of in vitro secondary mechanisms of genotoxicity induced by engineered nanomaterials. *Particle and fibre toxicology* 16: 8-14

Fahmy HM, Ebrahim NM, Gaber MH (2020) In-vitro evaluation of copper/copper oxide nanoparticles cytotoxicity and genotoxicity in normal and cancer lung cell lines. *Journal of trace elements in medicine and biology* 60: 126481

George JM, Magogotya M, Vetten MA, Buys AV, Gulumian M (2017) From the Cover: An Investigation of the Genotoxicity and Interference of Gold Nanoparticles in Commonly Used In Vitro Mutagenicity and Genotoxicity Assays. *Toxicological sciences* 156: 149

Guo X, Li Y, Yan J, Ingle T, Jones MY, Mei N, Boudreau MD, Cunningham CK, Abbas M, Paredes AM, Zhou T, Moore MM, Howard PC, Chen T (2016) Size- and coating-dependent cytotoxicity and genotoxicity of silver nanoparticles evaluated using in vitro standard assays. *Nanotoxicology* 10: 1373-1384

Haase A, Dommershausen N, Schulz M, Landsiedel R, Reichardt P, Krause B-C, Tentschert J, Luch A (2017) Genotoxicity testing of different surface-functionalized SiO2, ZrO2 and silver nanomaterials in 3D human bronchial models. *Archives of Toxicology* 91: 3991-4007

Hackenberg S, Scherzed A, Kessler M, Hummel S, Technau A, Froelich K, Ginzkey C, Koehler C, Hagen R, Kleinsasser N (2011a) Silver nanoparticles: Evaluation of DNA damage, toxicity and functional impairment in human mesenchymal stem cells. *Toxicology letters* 201: 27-33

Hackenberg S, Zimmermann F-Z, Scherzed A, Friehs G, Froelich K, Ginzkey C, Koehler C, Burghartz M, Hagen R, Kleinsasser N (2011b) Repetitive exposure to zinc oxide nanoparticles induces dna damage in human nasal mucosa mini organ cultures. *Environmental and molecular mutagenesis* 52: 582-589

Huk A, Izak-Nau E, el Yamani N, Uggerud H, Vadset M, Zasonska B, Duschl A, Dusinska M (2015) Impact of nanosilver on various DNA lesions and HPRT gene mutations - effects of charge and surface coating. *Particle and fibre toxicology* 12: 25

Jain AK, Singh D, Dubey K, Maurya R, Pandey AK (2019) Zinc oxide nanoparticles induced gene mutation at the HGPRT locus and cell cycle arrest associated with apoptosis in V‐79 cells. *Journal of applied toxicology* 39: 735-750

Kazimirova A, Baranokova M, Staruchova M, Drlickova M, Volkovova K, Dusinska M (2019) Titanium dioxide nanoparticles tested for genotoxicity with the comet and micronucleus assays in vitro, ex vivo and in vivo. *Mutation research Genetic toxicology and environmental mutagenesis* 843: 57-65

Kermanizadeh A, Løhr M, Roursgaard M, Messner S, Gunness P, Kelm JM, Møller P, Stone V, Loft S (2014) Hepatic toxicology following single and multiple exposure of engineered nanomaterials utilising a novel primary human 3D liver microtissue model. *Particle and fibre toxicology* 11: 56-56

Kim JS, Song KS, Sung JH, Ryu HR, Choi BG, Cho HS, Lee JK, Yu IJ (2013) Genotoxicity, acute oral and dermal toxicity, eye and dermal irritation and corrosion and skin sensitisation evaluation of silver nanoparticles. *Nanotoxicology* 7: 953-960

Kim Y-J, Yang SI, Ryu J-C (2010) Cytotoxicity and genotoxicity of nano-silver in mammalian cell lines. *Molecular & cellular toxicology* 6: 119-125

Kiratipaiboon C, Stueckle TA, Ghosh R, Rojanasakul LW, Chen YC, Dinu CZ, Rojanasakul Y (2019) Acquisition of cancer stem cell-like properties in human small airway epithelial cells after a long-term exposure to carbon nanomaterials. *Environmental Science: Nano* 6: 2152-2170

Královec K, Havelek R, Kročová E, Kučírková L, Hauschke M, Bartáček J, Palarčík J, Sedlák M (2019) Silica coated iron oxide nanoparticles-induced cytotoxicity, genotoxicity and its underlying mechanism in human HK-2 renal proximal tubule epithelial cells. *Mutation research Genetic toxicology and environmental mutagenesis* 844: 35-45

Kumbicak U, Cavas T, Cinkilic N, Kumbicak Z, Vatan O, Yilmaz D (2014) Evaluation of in vitro cytotoxicity and genotoxicity of copper–zinc alloy nanoparticles in human lung epithelial cells. *Food and chemical toxicology* 73: 105-112

Kung M-L, Hsieh S-L, Wu C-C, Chu T-H, Lin Y-C, Yeh B-W, Hsieh S (2015) Enhanced reactive oxygen species overexpression by CuO nanoparticles in poorly differentiated hepatocellular carcinoma cells. *Nanoscale* 7: 1820-1829

Kwon JY, Kim HL, Lee JY, Ju YH, Kim JS, Kang SH, Kim Y-R, Lee J-K, Jeong J, Kim M-K, Maeng EH, Seo YR (2014) Undetactable levels of genotoxicity of SiO2 nanoparticles in in vitro and in vivo tests. *International journal of nanomedicine* 9: 173-181

Kwon JY, Lee SY, Koedrith P, Lee JY, Kim K-M, Oh J-M, Yang SI, Kim M-K, Lee JK, Jeong J, Maeng EH, Lee BJ, Seo YR (2014) Lack of genotoxic potential of ZnO nanoparticles in in vitro and in vivo tests. *Mutation research Genetic toxicology and environmental mutagenesis* 761: 1-9

Landsiedel R, Ma-Hock L, Van Ravenzwaay B, Schulz M, Wiench K, Champ S, Schulte S, Wohlleben W, Oesch F (2010) Gene toxicity studies on titanium dioxide and zinc oxide nanomaterials used for UV-protection in cosmetic formulations. *Nanotoxicology* 4: 364-381

Li Y, Chen DH, Yan J, Chen Y, Mittelstaedt RA, Zhang Y, Biris AS, Heflich RH, Chen T (2012) Genotoxicity of silver nanoparticles evaluated using the Ames test and in vitro micronucleus assay. *Mutation Research/Genetic Toxicology and Environmental Mutagenesis* 745: 4-10

Li Y, Qin T, Ingle T, Yan J, He W, Yin J-J, Chen T (2017) Differential genotoxicity mechanisms of silver nanoparticles and silver ions. *Archives of toxicology* 91: 509-519

Llewellyn SV, Conway GE, Shah U-K, Evans SJ, Jenkins GJS, Clift MJD, Doak SH (2020) Advanced 3D Liver Models for In vitro Genotoxicity Testing Following Long-Term Nanomaterial Exposure. *Journal of Visualized Experiments*

Löbrich M, Shibata A, Beucher A, Fisher A, Ensminger M, Goodarzi AA, Barton O, Jeggo PA (2010) γH2AX foci analysis for monitoring DNA double-strand break repair: Strengths, limitations and optimization. *Cell Cycle* 9: 662-669

Manshian BB, Jenkins GJS, Williams PM, Wright C, Barron AR, Brown AP, Hondow N, Dunstan PR, Rickman R, Brady K, Doak SH (2013) Single-walled carbon nanotubes: differential genotoxic potential associated with physico-chemical properties. *Nanotoxicology* 7: 144-156

Mei N, Zhang Y, Chen Y, Guo X, Ding W, Ali SF, Biris AS, Rice P, Moore MM, Chen T (2012) Silver nanoparticle-induced mutations and oxidative stress in mouse lymphoma cells. *Environmental and molecular mutagenesis* 53: 409-419

Mrakovcic M, Meindl C, Leitinger G, Roblegg E, Fröhlich E (2015) Carboxylated short single-walled carbon nanotubes but not plain and multi-walled short carbon nanotubes show in vitro genotoxicity. *Toxicological sciences* 144: 114-127

Nymark P, Catalán J, Suhonen S, Järventaus H, Birkedal R, Clausen PA, Jensen KA, Vippola M, Savolainen K, Norppa H (2013) Genotoxicity of polyvinylpyrrolidone-coated silver nanoparticles in BEAS 2B cells. *Toxicology (Amsterdam)* 313: 38-48

OECD (2016a) *Test No. 473: In Vitro Mammalian Chromosomal Aberration Test*.

OECD (2016b) *Test No. 476: In Vitro Mammalian Cell Gene Mutation Tests using the Hprt and xprt genes*.

OECD (2016c) *Test No. 487: In Vitro Mammalian Cell Micronucleus Test*.

OECD (2016d) *Test No. 490: In Vitro Mammalian Cell Gene Mutation Tests Using the Thymidine Kinase Gene*.

OECD (2020) *Test No. 471: Bacterial Reverse Mutation Test*.

Paget V, Moche H, Kortulewski T, Grall R, Irbah L, Nesslany F, Chevillard S (2015) Human cell line-dependent WC-Co nanoparticle cytotoxicity and genotoxicity: A key role of ROS production. *Toxicological sciences* 143: 385-397

Reis ÉdM, Rezende AAAD, Santos DV, Oliveria PFd, Nicolella HD, Tavares DC, Silva ACA, Dantas NO, Spanó MA (2015) Assessment of the genotoxic potential of two zinc oxide sources (amorphous and nanoparticles) using the in vitro micronucleus test and the in vivo wing somatic mutation and recombination test. *Food and chemical toxicology* 84: 55-63

Sahu SC, Njoroge J, Bryce SM, Yourick JJ, Sprando RL (2014) Comparative genotoxicity of nanosilver in human liver HepG2 and colon Caco2 cells evaluated by a flow cytometricin vitromicronucleus assay. *Journal of Applied Toxicology* 34: 1226-1234

Valdiglesias V, Costa C, Kiliç G, Costa S, Pásaro E, Laffon B, Teixeira JP (2013a) Neuronal cytotoxicity and genotoxicity induced by zinc oxide nanoparticles. *Environment international* 55: 92-100

Valdiglesias V, Costa C, Sharma V, Kiliç G, Pásaro E, Teixeira JP, Dhawan A, Laffon B (2013b) Comparative study on effects of two different types of titanium dioxide nanoparticles on human neuronal cells. *Food and chemical toxicology* 57: 352-361

Wang X, Li T, Su X, Li J, Li W, Gan J, Wu T, Kong L, Zhang T, Tang M, Xue Y (2019) Genotoxic effects of silver nanoparticles with/without coating in human liver HepG2 cells and in mice. *Journal of applied toxicology* 39: 908-918

Wills JW, Hondow N, Thomas AD, Chapman KE, Fish D, Maffeis TG, Penny MW, Brown RA, Jenkins GJS, Brown AP, White PA, Doak SH (2015) Genetic toxicity assessment of engineered nanoparticles using a 3D in vitro skin model (EpiDerm™). *Particle and Fibre Toxicology* 13

Xia Q, Li H, Liu Y, Zhang S, Feng Q, Xiao K (2017) The effect of particle size on the genotoxicity of gold nanoparticles. *Journal of biomedical materials research Part A* 105: 710-719
